# Evidence of separate influence of moon and sun on light synchronization of mussel’s daily rhythm during the polar night

**DOI:** 10.1016/j.isci.2023.106168

**Published:** 2023-02-09

**Authors:** Damien Tran, Hector Andrade, Lionel Camus, Peter Leopold, Carl Ballantine, Jørgen Berge, Guillaume Durier, Mohamedou Sow, Pierre Ciret

**Affiliations:** 1 University of Bordeaux, EPOC, UMR 5805, 33120 Arcachon, France; 2 CNRS, EPOC, UMR 5805, 33120 Arcachon, France; 3 Institute of Marine Research, 9007 Tromsø, Norway; 4 Akvaplan-niva, Fram Centre for Climate and the Environment, 9296 Tromsø, Norway; 5 Faculty of Biosciences, Fisheries and Economics, UiT The Arctic University of Norway, 9037 Tromsø, Norway; 6 Centre for Autonomous Marine Operations and Systems, Department of Biology, Norwegian University of Science and Technology, Trondheim, Norway

**Keywords:** Biological sciences, Zoology, Evolutionary biology

## Abstract

Marine organisms living at high latitudes are faced with a light climate that undergoes drastic annual changes, especially during the polar night (PN) when the sun remains below the horizon for months. This raises the question of a possible synchronization and entrainment of biological rhythms under the governance of light at very low intensities. We analyzed the rhythms of the mussel *Mytilus sp*. during PN. We show that (1) mussels expressed a rhythmic behavior during PN; (2) a monthly moonlight rhythm was expressed; (3) a daily rhythm was expressed and influenced by both sunlight and moonlight; and (4) depending on the different times of PN and moon cycle characteristics, we were able to discriminate whether the moon or the sun synchronize the daily rhythm. Our findings fuel the idea that the capability of moonlight to synchronize daily rhythms when sunlight is not sufficient would be a crucial advantage during PN.

## Highlights

• During the polar night, *Mytilus sp*. expresses a monthly moonlight rhythm
• Both sunlight and moonlight synchronize the daily rhythm during the polar night
• Evidence of the separate moon and sun influences the mussel behavioral rhythm

## Graphical abstract

**Figure:**
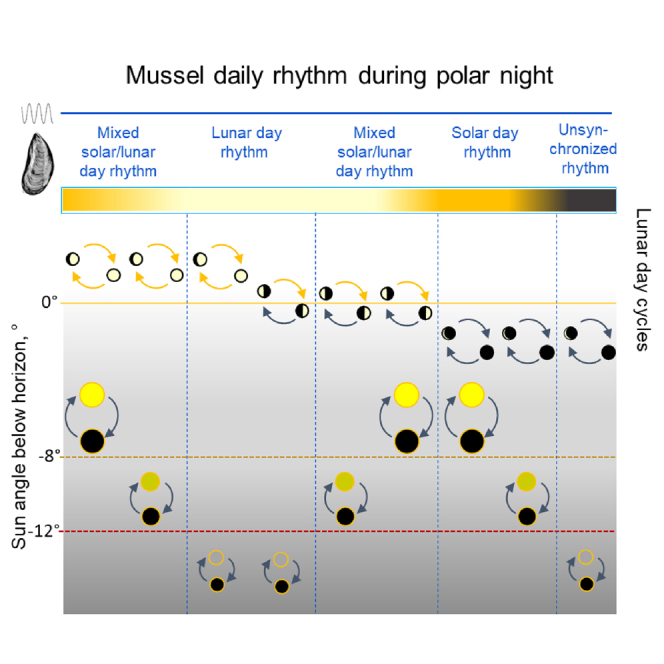

## Introduction

Recently, a habitual paradigm in Arctic marine biology stating that life processes are drastically reduced during the polar night (PN) have been challenged.^1^ Despite low temperature, limited food availability, and a reduction in illumination, PN dormancy does not appear to be a general feature among arctic marine organisms. Recent reviews have listed many active biological processes during the PN,^2^^,^^3^ among them being light-mediated biological rhythms expressed by zooplankton^4^^,^^5^^,^^6^ and an active daily rhythmic behavior in arctic scallops.^7^^,^^8^ Daily rhythms are usually synchronized by a 24-h solar-day cycle.^9^ Moreover, moonlight could also be involved in the lunar-month rhythm that is synchronized by a 29.5-day synodic moon cycle related to the moon surface illumination.^10^ It has been shown that Arctic zooplankton migrations can be mediated by lunar light during parts of the PN,^11^ but no studies have hitherto been able to document the absolute and relative influences of sun- and moonlight in relation to synchronizing biological rhythms during the PN. In the case of polar regions, and especially during the PN, the effect of sun and moon illumination to synchronize the rhythms are highly different from temperate areas where sun and moon cycles at the daily and monthly scales are mostly unchanged throughout the year. During PN, with the sun remaining below the horizon for a full 24-h cycle, light variation during the day still occurs, but in a very low range of intensity.^12^ At midday, the quantitative difference between the polar day and the polar night is in the order of 10^8^ measured in absolute quanta.^12^ Measured at 79°N within a diel cycle, the ratio of illumination between midday and midnight is approximately similar in the polar day and PN and six orders of magnitude lower than during the spring and autumn equinox.^12^^,^^13^ In polar regions, the moon cycles are also different from those observed at middle latitudes, due to the elliptic orbit of the earth around the sun. During a lunar month, we observe successively lunar-day cycles either always above or always below the horizon with between, as in temperate areas, cycles with the moon below and above the horizon each day. Moreover, the moon illumination is still changing at a 29.5-day cycle. At 79°N the moon light during a full moon, in the darkest part of the PN, is two orders of magnitude higher than the solar illumination (irradiance = ∼10^−3^ μmol m^−2^ s^−1^ and ∼10^−5^ μmol m^−2^ s^−1^, respectively) and hence the dominating factor regulating the lighting conditions.^12^^,^^13^ The blue mussel is a reemerged resident in the high Arctic archipelago of Svalbard after a 1000-year absence in Svalbard.^14^ On the archipelago, the mussel *Mytilus sp*. succeeded as a hybrid species, mixing three closely related species: *Mytilus edulis*, *Mytilus Galloprovincialis*, and *Mytilus trossulus*.^15^^,^^16^ This hybrid *Mytilus* species occupying high latitudes is faced with a new light climate, especially during PN, with the sun below the horizon for months. This raises the issue of a possible synchronization of biological rhythms under the governance of light at very low intensities and subsequent advantages.^17^^,^^18^^,^^19^^,^^20^^,^^21^^,^^22^^,^^23^^,^^24^ Thus, we have investigated if the light intensity from the sun and the moon during PN were sufficient to synchronize the behavioral rhythms of mussels and then, if it was possible to quantitatively separate the influences of the two.

## Results and discussion

### Synodic lunar-month cycles synchronize valve behavior of mussels during the polar night

**Figure 1:**
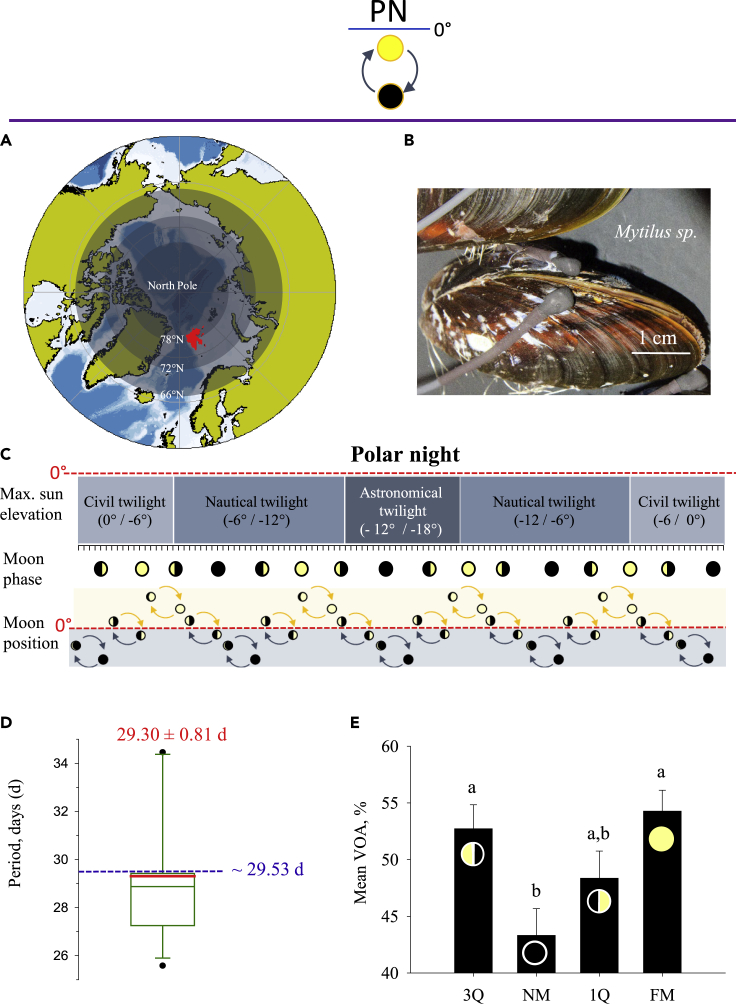
Moonlight rhythm of *Mytilus sp*. during the polar night in high Arctic (A) Map of the Kongsfjorden location, where the study was undertaken, a fjord in the Svalbard archipelago (in red), near Ny-Alesund (78° 56′ N, 11°56′ E). Different light regimes during PN: civil twilight between 66° and 72°N, nautical twilight between 72° and 78°N, and astronomical twilight between 72° and 78°N. (B) Details of a mussel equipped with electrodes of the HFNI valvometer biosensor to record its valve behavior. (C) Description of the studied polar night in Kongsfjorden (25/10/2017–17/02/2018, 116 days) according to (1) the maximum sun elevation leading to different twilight categories (civil, nautical, and astronomical) and (2) to lunar cycle comprising the moon phase (new moon , full moon , third quarter of the moon , first quarter of the moon ), and the moon position during a lunar-day cycle (moon above and below the horizon , moon always above the horizon ,, moon always below the horizon .). (D) Period analysis of valve activity rhythm determined by the spectral analysis Lomb-Scargle periodogram and validated by cosinor model during PN. Results are shown as quartiles in green (25% and 75% quartiles are defined by the box edge, 50% median value by the line inside the box). In red solid line, the mean. The exact mean value obtained is shown above in red (± SE, n = 15 mussels). Blue dotted line corresponds to the period of lunar month cycle lasting 29.53 days. (E) Mean daily VOA (valve opening amplitude, %) and SE(n = 15) according to the moon phases. 3Q and 1Q correspond to the third and first quarter of the moon respectively, NM: new moon and FM: full moon.  Pictogram that symbolizes sun daily cycle during PN always below the horizon. Identical letters indicate no significant differences of mean VOA (p value = 0.05).

To determine mussel’s rhythmic valve behavior during PN, we monitored valve opening amplitude (VOA) using a high-frequency non-invasive (HFNI) valvometer biosensor^25^ at Ny-Ålesund, in Kongsfjorden, Svalbard (78°56′ N, 11°56′ E) (Figure 1A). This biosensor has continuously recorded the behavior of 15 mussels equipped with valvometric electrodes (Figure 1B) during two consecutive PN (timetable in Table S1). To determine whether a moonlight rhythm was expressed, we analyzed PN 2016–2017 lasting 116 days. Figure 1C shows the different twilight intervals of the PN with corresponding successive moon phases and moon cycle positions according to the horizon. The valvometric data show clear moonlight rhythmic VOA. Individual rhythmic activities of VOA (Figure 1D) were determined by Lomb-Scargle periodogram spectral analysis^26^ to estimate the period and by the cosinor model^27^^,^^28^ to determine the characteristic and the strength of the rhythm. A significant period of 29.30 ± 0.81 days (mean ± ES) was found in the range of the synodic lunar-month cycle (∼29.53 days).. In Figure 1E, significant VOA differences were showed according to moon phase during PN (p = 0.012). VOA was minimal during new moon, intermediate during the first quarter of the moon, and maximal during full moon and the third quarter of the moon. A behavioral moonlight rhythm of a mollusk bivalve was already shown in the oyster *Crassostrea gigas* living in temperate areas. In winter, the daily rhythm of the *C*. *gigas* showed a maximal VOA during the new moon, the darkest time of the night.^25^^,^^29^ In contrast, we show here that during PN the mussels increase their VOA during the full moon, when moon light intensity is maximal. At an annual scale, we observed a higher VOA when the photoperiod and light intensity increased,^30^ suggesting a photophilic behavioral pattern. The influence of the lunar cycle during PN was previously reported for the vertical migration of pelagic zooplankton in the high Arctic.^11^ Thus, in this study, we show that eye-less benthic organisms such as *Mytilus sp*. have sensitivity and synchronicity toward the lunar cycle during PN.

### Mussels exhibit daily behavioral rhythms during the polar night

**Figure 2:**
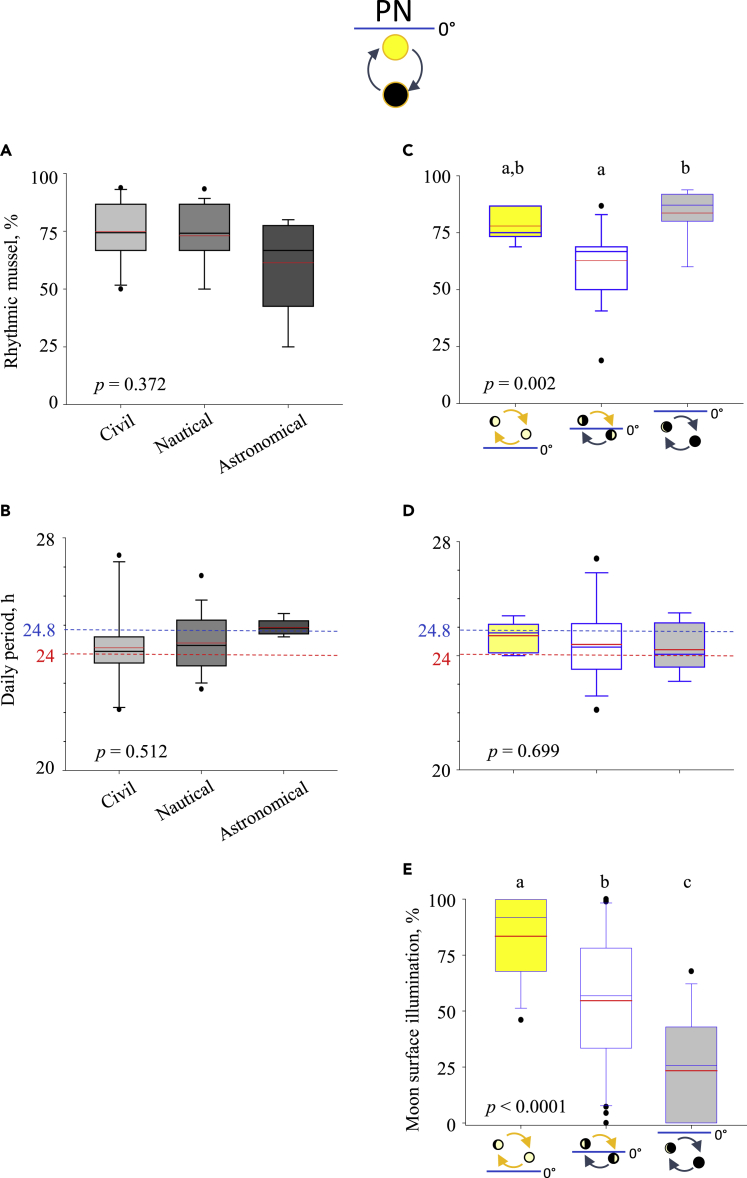
Daily behavioral rhythms of *Mytilus sp.* during the polar night according to the twilights or the moon cycles characteristics Period analysis of valve behavior (VOA) rhythm in the daily range (18-30 h) are determined by the spectral analysis Lomb and Scargle periodogram and validated by Cosinor model, during the two PN studied (see Figure S1 and Table S1). Results are shown as quartiles in green (25% and 75% quartiles are defined by the box edge, 50 % median value by the line inside the box). In red solid line, the mean. (A) Percentage of rhythmic mussels according to the different twilights of PNs: civil twilight (maximum sun height : 0° to −6°, n = 12 tested periods); nautical twilight (maximum sun height : −6° to −12°, n = 15 tested periods); astronomical twilight (maximum sun height : −12° to −18°, n = 4 tested periods). (B) Period analysis of daily valve activity rhythm according to the different twilights of PNs. (C) Percentage of rhythmic mussels according to the different lunar day cycles characteristics: lunar day cycles with the moon always above the horizon (yellow color, n = 7 tested periods); lunar day cycles with the moon above and below the horizon (white color, n = 16 tested periods); lunar day cycles with the moon always below the horizon (grey color, n = 8 tested periods). (D) Period analysis of daily valve activity rhythm according to the different lunar day cycles characteristics. (E) Moon surface illumination according to the different lunar day cycles characteristics. Significance of the pictograms and the tested intervals detailed in Table S2. Identical letters indicate no significant differences (*p*-value = 0.05).

To investigate whether light coming from lunar-day or solar-day cycles could synchronize valve behavior during PN, individual rhythmic activity of VOA on a daily scale was determined during two subsequent polar nights (details Table S1). Figure S1 shows the range of the daily rhythms measured during the two PN. These rhythms are calculated according to the different twilights, the moon phases, and the lunar cycle characteristics (31 periods studied in total, details Table S2). We showed that a significant rhythm was always expressed during PN regardless of the tested intervals. The percentage of rhythmic mussels ranges from 25% to 93.8% (see details Table S2). Moreover, the rhythms analyzed here showed periods ranging between 22.1 ± 1.1 h and 27.4 ± 0.5 h. It suggests a variable and quite weak synchronization of the sun or the moon at a daily scale during PN. That might be explained by the very low light intensity coming from the sun or the moon. To reveal a possible relationship between the light intensity of the sun reflected by the skyglow and the percentage of rhythmic mussels, we quantified percentages according to the different twilight intervals^31^^,^^32^^,^^33^ (Figure 2A). We show that the difference of light intensity in each twilight cannot explain the differences of percentage of rhythmic mussels alone. The results expressed as quartiles showed that around 73%–75% of mussels behaved rhythmic during civil and nautical twilights and ∼ 60% during the astronomical twilight (exact values in Table S3A). No significant difference (p = 0.372) was found between the three twilight conditions. These results suggested that a light cue coming from the sun is not able solely to entrain behavioral daily rhythms in mussels during PN. Then, we analyzed the percentages of rhythmic mussels (Figure 2B) and moon surface illumination (Figure 2C) according to the different lunar-day cycle characteristics. The results expressed a significant difference (p = 0.002) for rhythmic mussels. Indeed, during a lunar-day cycle with the moon above and below the horizon, the percentage of rhythmic mussels (62.8% ± 4.1%) was significantly lower compared with a lunar day with the moon always above the horizon (77.9% ± 2.6%) or always below the horizon (83.6% ± 3.8%). The mean illumination of the moon showed significant variation (p < 0.0001) between different moon cycles. The maximum percentage of illumination corresponds to different lunar day cycles. The mean illumination was 83.5% ± 5.2% with the moon always above the horizon, 54.7% ± 5.6% with the moon above and below the horizon, and 23.4% ± 6.0% with the moon always below the horizon. Considering both results (Figures 2B and 2C), we show that daily rhythm synchronization cannot be explained solely by lunar illumination or lunar cycle characteristics. Indeed, the percentage of rhythmic mussels was the same for lunar-day cycles, both always below and above the horizon, despite the variation of lunar disc illumination (exact values in Table S3B). Consequently, we conclude that the light cue coming from the moon is not sufficient by itself to explain behavioral rhythms at the daily scale. In conclusion, we show that daily rhythms of mussels are largely expressed during the polar night, but their synchronization cannot be explained separately by sunlight or moonlight cues.

### Both lunar day and solar day synchronize mussel’s daily rhythm during the polar night

**Figure 3:**
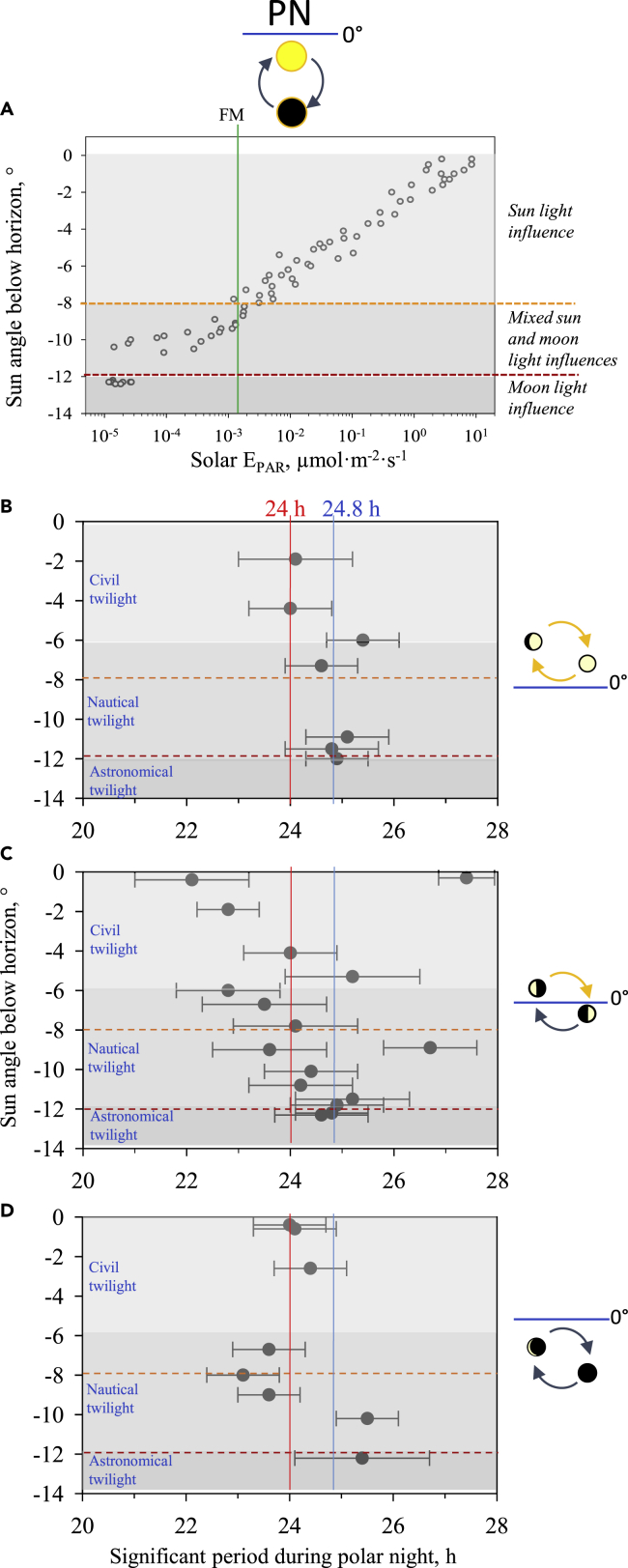
Daily behavioral rhythms of *Mytilus sp*. according to the sun elevation and the moon cycle characteristics (A) Solar E_PAR_ (μmol⸱m^−2^⸱s^−1^) at various solar altitudes below the horizon during PN. The graph is plotted using measured data published by Johnsen et al. 2021.^34^^,^^35^ Vertical solid green line corresponds to the full moon irradiance (∼ 1.5⸱10^−3^ μmol⸱m^−2^⸱s^−1^). Horizontal orange and dark orange dashed lines correspond to the sun elevation of −8° and −12°, respectively, which separated the sun and moon lights respective predominate influences. (B–D) Significant daily rhythms of VOA (mean ± ES, n = 15 mussels) during PN according to the maximal sun angle below the horizon and the moon cycle positions, determined by Lomb-Scargle periodogram and validated by cosinor analyses. (B) Lunar-day cycles with the moon always above the horizon (n = 7 tested periods); (C) lunar-day cycles with the moon above and below the horizon (n = 16 tested periods); (D) lunar-day cycles with the moon always below the horizon (n = 8 tested periods). For the details of the tested intervals, see Table S2. 24-h (solar-day) periodicity is defined by the red solid line, and 24.8-h (lunar-day) periodicity is defined by the vertical blue solid line.

By classic spectral analysis or rhythmic models, it is not possible to statistically discriminate the contribution of either sun cycle (24 h) or lunar cycle (24.8 h) to the mussel’s synchronization. Thus, to address the issue of the origin of the daily mussel rhythm during PN, we analyzed the evolution of the significant periods found in accordance to the interaction of light sources coming from the sun and the moon. First, to get an overview, in the Figure 3A, we plotted measured solar irradiance, expressed in energy of photosynthetic active radiation (E_PAR_), recorded in Ny-Alesund during PN^34^^,^^35^ according to its angle below the horizon. The maximal moonlight irradiance during full moon was measured at ∼1.5.10^−3^ μmol m^−2^ s^−1^, which also corresponded to a sunlight illuminance at an altitude of ∼−8° below the horizon.^12^^,^^32^ We were able to determine three different conditions that could be the zeitgeber of the daily rhythms. First, from 0° to −8° of sun elevation, only the sun could have a light influence. Then, in the range of −8° and −12° of sun elevation, sunlight and moonlight could have a mixed influence. And finally, below −12° of sun elevation (corresponding to the beginning of astronomical twilight), only the moon could have a light influence. To test the hypothesis that the evolution of the daily rhythms could reveal the different source of light synchronization, we plotted the significant periods found in the daily range during the two PN studied (Table S2) according to the sun elevation and the moon cycle positions (Figures 3B–3D). In the condition where the lunar-day cycle is always above the horizon (Figure 3B) and with a maximal moon surface illumination (Figure 2C), we show that the daily periods found were near 24 h in civil twilight, revealing a clear solely sun cycle influence and then moved toward periods close to a lunidian day at 24.8 h with the decrease of sun altitude to −12°. In the condition with the moon above and below the horizon during the lunar-day cycle (Figure 3C) and a moon surface with a mid-illumination (Figure 2C), we observe a higher variability of periods found in the daily range due to the mixed influence of the sun and the moon. But this variability decreased with the decrease of sun elevation to reach, at the end of the nautical twilight and the beginning of the astronomical one, daily periods closer to the lunidian cycle at 24.8 h. Finally, in the condition with the moon always below the horizon during the lunar-day cycle (Figure 3D) and a moon surface with a low illumination (Figure 2C), we clearly show that the periods found in the civil twilight up to the middle of the nautical twilight were close to 24 h under the influence of the sun. Then, at decreased sun elevation and lower lunar illumination, the periods found seem less synchronized by the sun or the moon, although a shift seem to occur to a period closer to 24.8 h rather than to 24 h.

### The strength of the moon entrainment to synchronize daily rhythm during the polar night depends on lunar-day cycle characterization, moon illumination, and the sun elevation

**Figure 4:**
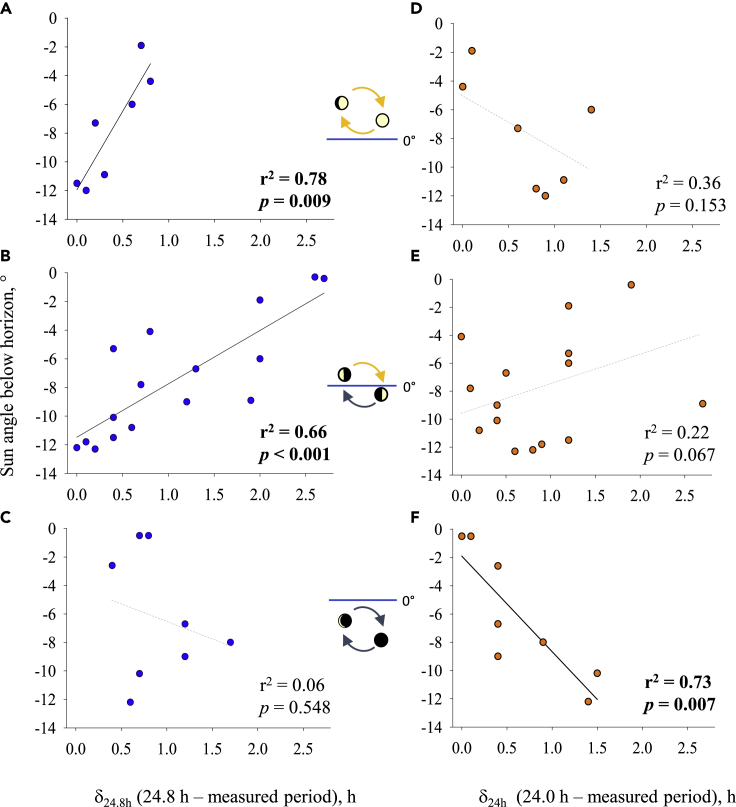
Entrainment strengths of the solar-day and lunar-day cycles on the behavioral rhythm accordingly to the interaction between the sun elevation and the moon cycle characteristics (A–F) Correlation between the sun angle position below the horizon and the difference in time duration (delta™) between lunar day (δ_24.8h_, (A–C)) or solar day (δ_24h_, (D–F)) and the actual periods found. Each correlation tested were done according to the moon cycle positions. (A, D) Lunar-day cycles with the moon always above the horizon (n = 7 tested periods); (B, E) lunar-day cycles with the moon cycles above and below the horizon (n = 16 tested periods), (C, F) lunar-day cycles with the moon always below the horizon (n = 8 tested periods). Lines solid black for significant and dotted gray for nonsignificant regressions. For the details of the tested intervals, see Table S2. In bolt, the significant p value of the linear regression and its corresponding r^2^.

To clarify and to order the respective influences of the moonlight and the sunlight in PN as the source entraining daily rhythms, we quantified the difference (δ) between the periods measured, with either the exact daily period of the sun cycle (24 h) or the daily period of the moon cycle (24.8 h). This parameter was used to evaluate the strength of entrainment of the moon and the sun, i.e. the lower the δ, the stronger the moon or sun influence. Figure 4 shows δ between a 24-h sun cycle (δ_24h_) or 24.8-h moon cycle (δ_24.8h_) and the actual periods found according to the moon cycle characteristics and the sun elevation. We can show a significant correlation between δ_24.8h_ and sun elevation, for conditions when the moon cycle was always above the horizon (p = 0.009, r^2^ = 0.78) and moon cycle above and below the horizon (p < 0.001, r^2^ = 0.66) (Figures 4A–4B). In these lunar conditions, the lower the sun elevation, the more δ_24.8h_ became closer to zero. On contrary, under these lunar conditions, we did not find a significant correlation between δ_24h_ and sun elevation (Figures 4D–4E). These results highlight that moonlight was the main driver of the daily rhythm under these conditions. Then, we showed (Figure 4F) a significant correlation between δ_24h_ and sun elevation, when the moon cycle was always below the horizon (p = 0.007, r^2^ = 0.73). In this condition, the higher the sun elevation, the more δ_24h_ approached zero. On contrary, we did not observe a significant correlation between δ_24.8h_ and sun elevation (Figure 4C), showing an absence of moon influence to synchronize the daily rhythm.

### During the polar night, moonlight and sunlight share the timing of daily rhythm

We have focused on the mollusk bivalve *Mytilus sp*. to investigate the role of the moonlight during PN in the synchronization of daily behavior. We have shown that moonlight, besides its role as a driver of monthly rhythm, also shares the role of timing daily behavior together with the sunlight. Usually, it is assumed that the daily rhythm is the consequence of the circadian clock synchronization by sunlight. However, during PN conditions, moonlight intensity is comparable to sunlight and could set the mussel’s daily timing system.

Our finding does not allow to discriminate if the observed daily activity is due to a direct reaction to moonlight/sunlight perception or is due to endogenous clockwork mechanism entrainment. However, a previous study done on the arctic scallop *Chlamys islandica* in Svalbard^8^ showed the first evidence of the persistence of clock gene expression oscillations during PN, suggesting that functional clockwork could entrain rhythmic behaviors. Moreover, earlier studies^36^^,^^37^ on bivalves such as the oyster *C*. *gigas* have highlighted the plasticity of the so-called circadian clock that was able to run at tidal periodicity (∼12.4 h) under field conditions and under constant darkness in controlled lab conditions, whereas this clock ran at ∼24 h under light/dark conditions. Although our finding strongly suggests a photophilic behavior of the mussels in PN, it does not prove it *stricto sensu*. Indeed, there may exist an indirect effect through the interaction with the food sources or predators that would be under the synchronization of the lunar cycles. However, evidence that the circadian clock could be sensitive to low light intensity in the range of moonlight have already been shown in different terrestrial animals^38^^,^^39^ and plants.^40^ This plasticity of the circadian clock has also been suggested for other marine phyla. Molecular work on the bristleworm *Platynereis dumerilii* revealed that moonlight is perfectly able to synchronize swarming daily rhythm thanks to the interplay of two light sensors, a melanopsin ortholog (R-opsin1) and a cryptochrome (L-cry), known to be involved in the circadian clockwork.^41^ Finally, if we accept the idea that maintaining daily rhythm during PN is crucial for animals to temporally prioritize internal physiological processes, the ability to synchronize the clock system by moonlight when sunlight is not sufficient would be a key advantage for animals in polar regions.

### Limitations of the study

Although we measured an apparent light-mediated rhythm, we are not able to disentangle a direct response to light of the mussels from a response controlled by an endogenous underpinning clockwork mechanism. Moreover, in this field experiment, we did not strictly prove the photophilic behavior of the mussels. Thus, only the expression of clock genes in the same conditions would allow to give an answer about the photophilic behavior and the involvement of the clock in the generation of the behavioral rhythm in such low light intensity and also the implication of the circadian clock on the light perceived from the moon. Thus, a molecular study remains to be achieved to complete the answer of this issue.

## STAR★Methods

### Key resources table

**Table:** 

REAGENT or RESOURCE	SOURCE	IDENTIFIER
**Devices**		

HFNI valvometer biosensor	Laboratory- made	damien.tran@u-bordeaux.fr
Valvometer electrodes	Imprelec manufacturer	32 Rue de l'Égalité 39360 Viry, France
Valvometer electronic cards	Nanog manufacturer	http://www.nanog.fr/

**Deposited data**		

Raw and analyzed data	This paper	Data S1 and S2

**Experimental models: Organisms/strains**		

Mollusk bivalves, blue mussel (Mytilus *sp*.)	Isfjorden, Svalbard	N/A

**Software and algorithms**		

TSA-Serial Cosinor 8.0 package	Expert Soft Tech	http://www.euroestech.net/mainfr.php
SigmaPlot 13.0	Systat Software, Inc	https://systatsoftware.com/products/sigmaplot/
Labview 8.0	National instruments	http://www.ni.com/fr-fr/shop/labview.html

### Resource availability

#### Lead contact

Further information and requests for resources and reagents should be directed to and will be fulfilled by the lead contact, Damien Tran (damien.tran@u-bordeaux.fr).

#### Materials availability

This study did not generate new unique reagents. All key resources are listed in the key resources table. Further information and requests for resources and reagent should be directed to the lead contact.

### Experimental model and subject details

All animal studies were conducted in accordance with local legislation. All investigations were performed on mussels, *Mytilus sp*.. All mussels were collected manually by a diver from natural recruitment in Isfjorden, near Longyearbyen (latitude 78°13′N, longitude 15°38′E), Svalbard. Before experiment, mussels were translocated and acclimated for 2 months at the experimental site.

### Method details

#### Field study and experimental conditions

Field experiments were performed at Ny-Alesund in Kongsfjorden, Svalbard (latitude 78° 56′ N, 11° 56′ E), on the western coast of the Spitsbergen Island. Mussels were placed at the seafloor in a ballasted cage (50 × 50 × 100 cm) at a depth of 3 m (±tides), always in subtidal conditions, under an old pier in Ny-Alesund. This study was conducted on 15 mussels (56.3 ± 3.7 mm shell length) and performed over a 332-day period, including two PN (i.e. period of the year in polar regions where the sun is always below the horizon) lasting 4 months each, see detailed timetable in Table S1. The astronomical data related to sun, earth, and moon positions were retrieved from the website www.timeanddate.com and are available in Table S1.

#### Light climate at Ny-Alesund

The quantification of sunlight climate at Ny-Alesund was performed using measured data from Johnsen et al.^34^^,^^35^ recorded during the polar night 2017–2018 (24/10/2017-17/02/2018).

Solar irradiance was expressed in energy of photosynthetic active radiation (E_PAR_, μmol⸱m^−2^⸱s^−1^). We plotted E_PAR_ data measured at midday (12h UTC). To avoid light coming from the moon, for data with sun angle below −8° (when moon light becomes influent), only data with moon below the horizon and/or with the percentage of lunar disc illumination <10% were considered.

#### Recording of mussel’s valve activity behavior in the field study

Valve behavior was recorded *in situ* using a high-frequency non-invasive (HFNI) valvometer.^25^ Briefly, a pair of lightweight electrodes, designed to minimize disturbance to mussel’s behavior, were glued on each shell. These valvometer electrodes were connected to the HFNI valvometer by flexible wires, which allowed the mussels to move their valves without constraints. The measurement is magnetic principle based. The electrodes are made with small self-inductance solenoids (material: ferrite; size: 3.2 mm × 2.5 mm x 2 mm; weight: ∼0.06 g), whose specifications are: inductance: 470 μH; rated current: 45 mA; self-resonance frequency: 5 mHz. Thanks to these electrodes specificity, a very low electromagnetic current (1–2 nT) was generated between the electrodes by the biosensor, which allowed measurements of the amount of valve opening. The signal was recorded at 10 Hz using custom acquisition cards (Nanog manufacturer, Pessac, France), and the data were automatically transmitted daily to a data processing center at the Arcachon Marine Biological Station (France) using internet network.

### Quantification and statistical analysis

#### Valve behavior quantification

Field valve activity data were analyzed using LabView 8.0 software (National Instruments). The valve behavior endpoints were expressed as the hourly valve opening amplitude (VOA, %) of each individual (54,000 data acquisition/day/mussel). Mean hourly VOA was reported as a percentage, with 100% indicating that the valves were opened at their maximum amplitude and 0% indicating that the valves were closed, during the entire time studied, respectively.

#### Chronobiological analysis

##### Determination of tested periods for chronobiological analysis

To determine the existence of individual mussel’s lunar month rhythm based on the synodic lunar cycles lasting 29.53 days, chronobiological analysis were applied on the 2017–2018 PN-period, comprising 4 entire synodic lunar cycles. Such chronobiological analysis couldn’t be done in the first PN studied (2016–2017) at the monthly scale due to the absence of data during 15 days during this time due to electrical failures. Lunar month periodicity of behavioral rhythm was defined significant for a period of 29.53days ±5 days. To calculate VOA according to the moon phases (full moon, first quarter of the moon, new moon and third quarter of the moon), we measured for each mussel the mean of VOA during 3 days around the exact date of each moon phase, and so for each of the 4 lunar-month cycles comprised in PN.

Then, we determined daily rhythms. In this study, we used the term daily for all periods found in the range 18-30h, entrained by a 24h sun daily cycle (solar day) or a 24.8h lunar daily cycle (lunidian day). The extent of the classic circadian range (20-28h) previously defined for terrestrial animals was set to consider possible looser periodicity in unsynchronized conditions or weakly synchronized for aquatic organisms in PN.

To determine rhythm at the daily scale, the two PN were analyzed. These two PN were divided in several intervals according to the lunar day cycles (see details Tables S1 and S2).

##### Methods to quantify significant rhythms

Chronobiological analyses were performed using TSA Serial Cosinor 8.0 software. Several steps were required to validate a significant rhythm.^42^^,^^43^ Four steps must be validated. First, the quality of the dataset was assessed by controlling for the absence of randomness using the autocorrelation diagram. Second, the absence of a stationary phenomenon was checked by using a partial autocorrelation function (PACF) calculation.^44^ Third, the recorded data were tested for periodicities by the spectral method of the Lomb and Scargle periodogram, which combines the principle of a regression analysis and Fourier transformations.^26^ This method gives a threshold of probability (p = 0.95) defining the limit below which the signal can be regarded as “noise”. Fourth, the rhythmicity was validated and modeled with the cosinor model, which uses a cosine function calculated by regression.^27^^,^^28^ For a given period, the model is written as Y (t) = Acos (πt/τ + φ) +M + ε (t) where Y (t) is an observation of the mean VOA at time t, A is the amplitude, φ is the acrophase, τ is the period, M is the mesor and ε is the relative error. Two key tests validated the calculated model and the existence of a rhythm: the elliptic test had to be rejected, and the probability for the null amplitude hypothesis had to be <0.05. For a set of data, several significant periodicities could occur. To identify significant secondary periodicities, we reinjected the previously calculated residues of the Cosinor model to remove the trend related to the first statistical period and then repeated the entire procedure (1–4 steps). This entire procedure was necessary to validate secondary periodicities. In this study, the procedure was repeated up to four times to reveal significant rhythmicity.

##### Statistical analysis

Differences between conditions were investigated using one-way ANOVA for repeated measures, after checking assumptions (normality of data and equal variance tests). When assumptions were not validated, non-parametric tests were performed. A Kruskal–Wallis One-Way ANOVA on rank for repeated measures was applied to compare distributions, followed by Dunn’s Method for all pairwise multiple comparisons. For all statistical results, a probability of p < 0.05 was considered significant. Analyses were performed with SigmaPlot (Version 13, Systat, Chicago, USA).

## Data Availability

• All data are available in Data S1 and S2 files. • This paper does not generate original code. • Any additional information required to reanalyze the data reported in this paper is available from the lead contact upon request.

## Supplemental information

Document S1. Figure S1 and Tables S1–S3

Data S1. Dataset of hourly valve opening amplitude data during the polar night 2016–2017, related to STAR Methods

Data S2. Dataset of hourly valve opening amplitude data during the polar night 2017–2018, related to STAR Methods
