# Supplementary material for: Evidence of separate influence of moon and sun on light synchronization of mussel’s daily rhythm during the polar night
Source: iScience. 2023 Feb 9;26(3):106168. doi: 10.1016/j.isci.2023.106168 (PMC9978622; doi:10.1016/j.isci.2023.106168)
Supplement: Document S1. Figure S1 and Tables S1–S3 [file mmc1.pdf]

iScience, Volume *26*

Supplemental information

Evidence of separate influence of moon and sun on light synchronization of mussel's daily rhythm during the polar night

Damien Tran, Hector Andrade, Lionel Camus, Peter Leopold, Carl Ballantine, Jørgen Berge, Guillaume Durier, Mohamedou Sow, and Pierre Ciret

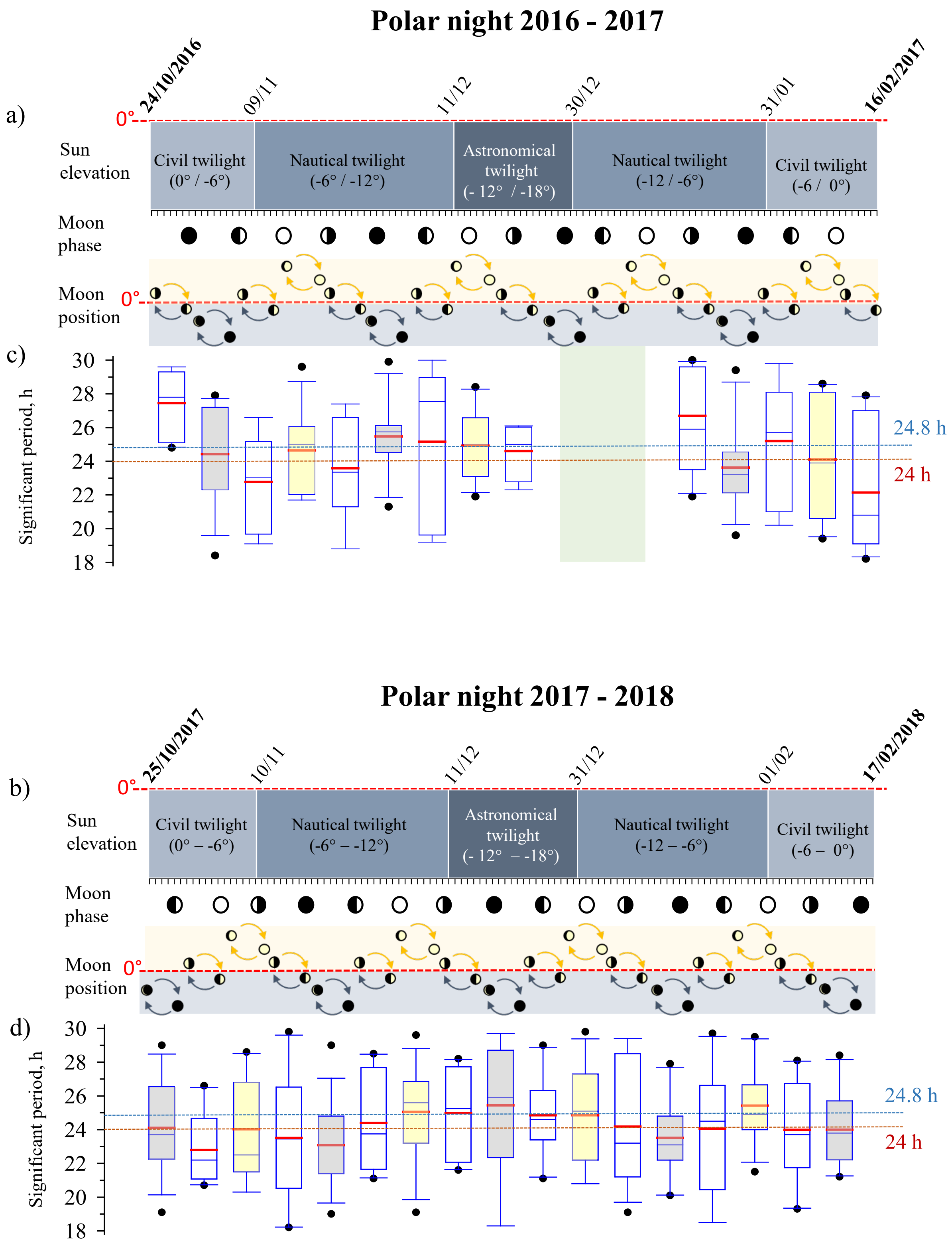

Figure S1_Tran et al.

Figure S1. Daily rhythm of *Mytilus sp.* during two polar night in high Arctic, related to Figure 2. (a - b) Description of the two studied polar night (PN) in the Kongsforden (PN1: 24/10/2016 – 16/02/2017; PN2: 25/10/2017 – 17/02/2018) according to 1) the maximum sun elevation leading to different twilight categories (civil, nautical and astronomical) and 2) to lunar cycle comprising the moon phase (new moon ● , full moon ○ , third quarter of the moon ◐ , first quarter of the moon ◑), and the moon position during a lunar-day cycle (moon always above the horizon , moon above and below the horizon , moon always below the horizon). **(c - d)** Period analysis of daily valve activity rhythm determined by the spectral analysis Lomb and Scargle periodogram and validated by Cosinor model, respectively during PN1 and PN2. Results are shown as quartiles in blue (25% and 75% quartiles are defined by the box edge, 50 % median value by the line inside the box). In red solid line, the mean. The exact mean value obtained (± SE, n = 15 mussels) is given in the Table S2. Blue dotted line corresponds to the period of the lunidian day lasting 24.8 h and the red dotted line correspond to the period of the solar day lasting 24 h.

Table S1. Timetable of the twilight periods during the studied polar nights, related to Figure 2. Dates of the different twilights during the two polar nights studied in Kongsfjorden (Spitsbergen Island, Svalbard; 78° 56' N, 11° 56' E), near Ny-Alesund. 15 days of data are missing due to electrical failure.

Twilight periods *(sun elevation)*	**Polar night 1 2016-2017** (116 days)	**Polar night 2 2017-2018** (116 days)	*Missing data*
Civil 1 *(0° to -6°)*	24/10/16 - 09/11/16 (*17 d*)	25/10/17 – 09/11/17 (*16 d*)	
Nautical 1 *(-6° to -12°)*	10/11/16 – 11/12/16 (*32 d*)	10/11/17 – 11/12/17 (*32 d*)	
Astronomical *(-12° to -18°)*	12/12/16 – 30/12/16 (*19 d*)	12/12/17 – 30/12/17 (*19 d*)	26/12/16 – 30/12/16 (*5 d*)
Nautical 2 *(-12° to -6°)*	31/12/16 – 31/01/17 (*32 d*)	31/12/17 – 31/01/18 (*32 d*)	31/12/16 – 09/01/17 (*10 d*)
Civil 2 *(-6° to 0°)*	01/02/17 – 16/02/17 (*17 d*)	01/02/18 – 17/02/18 (*18 d*)	

Table S2. Tested periods according to sun elevation and lunar day cycles during polar nights, related to Figure 3. Chronobiology analysis done with Lomb and Scargle periodogramm combined to Cosinor method.

A - **Polar night 2016 – 2017**

Period dates	Tested days, n	Hourly VOA analysed/ind.	Sun elevation (max.)	Lunar day cycles	Moon surface illumination (%)	Rhythmic ind. (%)	Significant rhythms, h
24/10/16 – 30/10/16	6 d	144 h	-0.3 °	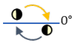	0 - 34.9	68.8	27.4 ± 0.5
31/10/16 – 08/10/16	9 d	216 h	-2.6 °	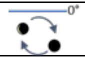	0.7 - 59.8	93.8	24.4 ± 0.7
09/11/16 – 13/11/16	5 d	120 h	-6.0 °	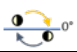	70.5 - 99.2	50	22.8 ± 0. 9
14/11/16 – 20/11/16	7 d	168 h	-7.3 °	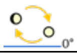	60.0 - 100	75	24.6 ± 0.7
21/11/16 – 26/11/16	6 d	144 h	-9.0 °	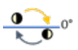	8.8 – 51.6	50	23.6 ± 1.1
27/11/16 – 05/12/16	9 d	216 h	-10.2 °	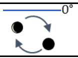	0 – 32.4	87.5	25.5 ± 0.6
06/12/16 – 11/12/16	6 d	144 h	-11.5 °	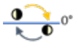	42.9 – 93.1	50	25.2 ± 1.7
12/12/16 – 18/12/16	7 d	168 h	-12.0 °	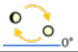	78.7 - 100	75	24.9 ± 0.6
19/12/16 – 24/12/16	6 d	144 h	-12.3 °	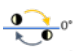	21.2 - 69.1	25	24.6 ± 0.9
25/12/16 – 01/01/17	8 d	192 h	-11.9 °	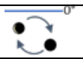	0 – 13.9	*6 days missing*	NS
02/01/17 – 07/01/17	5 d	120 h	-11.2 °	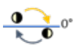	18.0 – 71.8	*5 days missing*	NS
08/01/17 – 14/01/17	7 d	168 h	-10.1 °	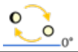	82.0 - 100	*4 days missing*	NS
15/01/17 – 20/01/17	6 d	144 h	-8.9 °	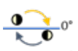	47.3 - 91.7	68.8	26.7 ± 0.9
21/01/17 – 29/01/17	9 d	216 h	-6.7 °	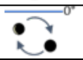	0 – 37.7	87.5	23.6 ± 0.7
30/01/17 – 03/02/17	5 d	120 h	-5.3 °	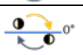	7.2 – 44.7	50	25.2 ± 1.3
04/02/17 – 11/02/17	8 d	192 h	-1.9 °	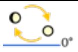	56.5 - 100	68.8	24.1 ± 1.1
12/02/17 – 16/02/17	5 d	120 h	-0.4 °	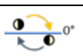	74.5 – 98.9	81.3	22.1 ± 1.1

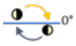 Lunar day with moon below and above the horizon during the 24.8 cycle

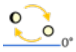 Lunar day with moon always above the horizon

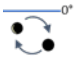 Lunar day with moon alway below the horizon

B - **Polar night 2017 - 2018**

Period dates	Tested days, n	Hourly VOA analysed/ind.	Sun elevation (max.)	Lunar day cycles	Moon surface illumination (%)	Rhythmic ind. (%)	Significant rhythms, h
25/10/17 – 29/10/17	5 d	120 h	-0.5 °	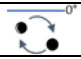	29.1-67.8	86.7	24.1 ± 0.8
30/10/17 – 03/11/17	5 d	120 h	-1.9 °	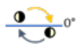	77.2 - 100	66.7	22.8 ± 0.6
04/11/17 – 11/11/17	8 d	192 h	-4.4 °	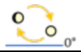	46.0 – 99.0	86.7	24.0 ± 0.8
12/11/17 – 16/11/17	5 d	120 h	-6.7 °	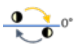	4.4 – 35.0	66.7	23.5 ± 1.2
17/11/17 – 26/11/17	10 d	240 h	-8.0 °	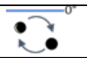	0 – 50.2	93.3	23.1 ± 0.7
27/11/17 – 01/12/17	5 d	120 h	-10.1 °	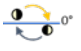	60.6 – 95.2	66.7	24.4 ± 0.9
02/12/17 – 08/12/17	7 d	168 h	-10.9 °	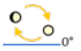	73.3 - 100	86.7	25.1 ± 0.8
09/12/17 – 14/12/17	6 d	144 h	-11.8 °	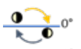	13.7 – 62.5	66.7	24.9 ± 0.9
15/12/17 – 23/12/17	9 d	216 h	-12.2 °	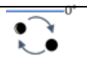	0 – 23.8	60.0	25.4 ± 1.3
24/12/17 – 28/12/17	5 d	120 h	-12.2 °	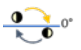	32.9 – 75.1	80	24.8 ± 0.7
29/12/17 – 05/01/18	8 d	192 h	-11.5 °	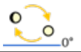	84.7 - 100	73.3	24.8 ± 0.9
6/01/18 – 10/01/18	5 d	120 h	-10.8 °	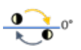	37.0 – 78.5	86.7	24.2 ± 1.0
11/01/18 – 19/01/18	9 d	216 h	-9.0 °	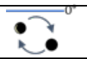	0 – 27.6	80	23.6 ± 0.6
20/01/18 – 25/01/18	6 d	144 h	-7.8 °	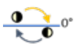	10.9 – 59.1	66.7	24.1 ± 1.2
26/01/18 – 01/02/18	7 d	168 h	-6.0 °	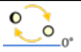	70.3 - 100	80	25.4 ± 0.7
02/02/18 – 07/02/18	6 d	144 h	-4.1 °	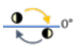	54.7 – 96.9	66.7	24.0 ± 0.9
08/02/18 – 16/02/18	9 d	216 h	-0.5 °	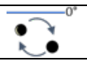	0 – 44.6	80	24.0 ± 0.7

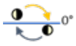 Lunar day with moon below and above the horizon during the 24.8 cycle

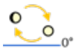 Lunar day with moon always above the horizon

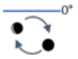 Lunar day with moon alway below the horizon

Table S3. Rhythmic mussels in the daily range during the polar night, related to Figure 2.

A. According to the different twilights. **B.** According to the lunar day cycles characteristics and the mean of moon surface illumination. Chronobiology analysis done with Lomb and Scargle periodogramm combined to Cosinor method.

A.

Twilights	Rhythmic mussels, %	Number of tested periods during PN
Civil	74.9 ± 4.1 %	10
Nautical	73.1 ± 3.6 %	16
Astronomical	61.3 ± 9.7 %	5

B.

Moon cycles during PN	Symbols	Moon surface illumination. Mean ± ES	Rhythmic mussels, %	Number of tested periods during PN
Always above the horizon	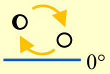	83.5 ± 5.2 %	77.9 ± 2.6 %	7
Alternation above and below the horizon	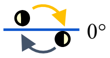	54.7 ± 5.6 %	62.8 ± 4.1 %	16
Always below the horizon	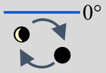	23.4 ± 6.0 %	83.6 ± 3.8 %	8
